# Gynecologic Care of Black Breast Cancer Survivors

**DOI:** 10.1007/s12609-024-00527-4

**Published:** 2024-02-19

**Authors:** Versha Pleasant

**Affiliations:** 1 University of Michigan Hospital, Mott Children & Women’s Hospital, 1500 East Medical Center Drive, Ann Arbor, MI 48109, USA

**Keywords:** Breast cancer, Black race, Gynecology, Racial health disparities

## Abstract

**Purpose of Review:**

Black patients suffer from breast cancer-related racial health disparities, which could have implications on their gynecologic care. This review explores considerations in the gynecologic care of Black breast cancer survivors.

**Recent Findings:**

Black people have a higher risk of leiomyoma and endometrial cancer, which could confound bleeding patterns such as in the setting of tamoxifen use. As Black people are more likely to have early-onset breast cancer, this may have implications on long-term bone and heart health. Black patients may be more likely to have menopausal symptoms at baseline and as a result of breast cancer treatment. Furthermore, Black patients are less likely to utilize assisted reproductive technology and genetic testing services.

**Summary:**

It is important for healthcare providers to be well-versed in the intersections of breast cancer and gynecologic care. Black breast cancer survivors may have unique considerations for which practitioners should be knowledgeable.

## Introduction

Breast cancer represents the most commonly diagnosed cancer among people assigned female at birth (AFAB) in the United States and the second leading cause of cancer death among this group [1]. Despite its prevalence, breast cancer tends to be diagnosed at stage I and carries a 90% 5-year survival [2]. However, these outcomes differ drastically with regard to race. Based on data from the American Cancer Society, Black people have similar rates of breast cancer compared to White people (127.8 per 100,000 versus 133.7 per 100,000, respectively) but have a 40% increased breast cancer mortality compared to White people [3]. Black communities have the lowest survival compared to all other racial groups across all stages and subtypes of breast cancer. While increased diagnosis of more aggressive subtypes such as triple-negative breast cancer (TNBC) plays a role in survival, the depth of breast cancer-related mortality among Black people goes far beyond hormone receptors. It represents a complex interplay of clinical risk factors abutting social determinants of health—all within the context for which systemic racism is woven into the very fabric of the healthcare system.

While most breast cancer patients are under the care of a medical oncologist during active treatment, breast cancer survivors are still in need of general medical care. Many patients may present to their gynecologists for routine wellness exams or may have questions about how breast cancer relates to their gynecologic screenings. Given the disproportionate impact of breast cancer on the Black community, Black patients may have unique needs and considerations regarding their gynecologic care. This article focuses on the intersection of breast cancer and gynecologic care among Black communities, the highlights of which are summarized in Table 1.

## Routine Gynecologic Care

Cancer can be a frightening term, and often breast cancer survivors may feel that their global cancer risk increases after having had breast cancer. Patients should generally be reassured that they should continue with their routine gynecologic screenings (except for those with a strong family history of cancer and/or those who have had positive germline testing for hereditary breast and ovarian cancer syndromes).

### Breast Cancer Screening

Mammography is an effective screening tool and has been proven to decrease mortality [4]. Most medical societies recommend age of initiation at 40 years old for average risk individuals [5–8]. Despite conflicting recommendations regarding screening intervals, medical societies such as the National Comprehensive Cancer Network (NCCN) and the American College of Radiology (ACR) recommend screening mammography annually [6, 9].

Regarding screening for breast cancer survivors, NCCN guidelines state that screening mammograms should continue every 12 months after a breast cancer diagnosis for those with intact breast tissue [10]. ACR recommends that patients diagnosed with breast cancer < 50 years old or with a personal history of breast cancer and dense breasts should undergo supplemental screening with contrast-enhanced breast magnetic resonance imaging (MRI) [11••]. For those with known germline pathogenic mutations such as BRCA1/2, practitioners should follow NCCN guidelines regarding alternating mammogram and breast MRI for patients with intact breast tissue.

There are no established guidelines specifically regarding early onset Black breast cancer survivors and subsequent breast cancer screening. However, practitioners should recognize that breast cancer recurrence is more common in Black communities, as well as contralateral breast cancer after the initial primary [12–14]. One study also demonstrated an increased prevalence of dense breast tissue among Black people compared to White people [15], which is known to be an independent risk factor for breast cancer [16, 17]. Despite practitioners being required to inform patients of the presence of dense breast tissue, there is no current national consensus on supplemental screening for those whose sole risk factor is dense breast tissue in the absence of other risk factors. Given these multiple risk factors, practitioners should stress routine screenings even in light of a prior breast cancer diagnosis.

Generally, screening imaging is not indicated for those who have undergone bilateral mastectomies; screening in these cases consists of self “breast” awareness by the patient and annual chest wall exam by the healthcare provider. Imaging may be indicated for diagnostic purposes or for evaluation of implant integrity.

### Cervical Cancer Screening

Current data suggests that a major contributor to cervical cancer risk is infection with human papillomavirus (HPV), which is a common sexually transmitted infection. Therefore, there is no data suggesting an increased risk of cervical cancer solely due to a personal history of breast cancer. The rare exception to this is those who carry germline pathogenic mutations in STK11 (also called Peutz-Jeghers syndrome), which is associated with an increased lifetime risk of breast cancer and minimal deviation adenocarcinoma of the cervix (also known as adenoma malignum) [18, 19].

Therefore, pap smears should be performed at routine intervals as recommended as per the American Society for Colposcopy and Cervical Pathology (ASCCP) [20], which is in accordance with guidelines from the United States Preventive Services Task Force (USPSTF). These guidelines recommend: cervical cancer screening between the ages of 21 and 29 every 3 years with cytology alone, and for ages 30–65 either every 3 years with cytology alone, every 5 years with high-risk HPV testing alone, or every 5 years with cytology and high-risk HPV co-testing (grade A recommendations). These guidelines should be consulted regarding management of those with abnormal pap smears, precancerous lesions, and other unique considerations, which are outside of the scope of this article.

It should be noted that although screening schedules need not be modified solely as a result of a breast cancer diagnosis due to the aforementioned reasons, Black people are disproportionately affected by cervical cancer. The incidence rate is 8.8 per 100,000 and 7.2 per 100,000 for Black and White people, respectively, with a 22% increased incidence rate among Black people. Regarding mortality, Black people are 65% more likely to die from cervical cancer compared to White people, which is expected to be an underestimate due to the high prevalence of hysterectomy among Black communities [21]. This makes screening critical in this community and should be prioritized as a component of routine care.

### Other GYN Cancer Screening

As is the case in the general population, there is no routine screening for ovarian or endometrial cancers. Breast cancer survivors should be reassured that they do not need to initiate any new screenings in this regard (again, except for those with a germline pathogenic variant or in some cases for those with family history). While historical gynecologic care has entailed “routine” or “annual” pelvic exams, more recent guidelines provide a new perspective on a historically common practice. According to the American College of Obstetricians and Gynecologists (ACOG), pelvic exams should be performed primarily for medical history or symptoms. Due to the possibility of harm and little data showing utility in asymptomatic patients, shared-decision making should be employed between the patient and practitioner [22].

Patients may inquire if oophorectomy is indicated in the setting of a breast cancer diagnosis. Increased ovarian cancer risk should not necessarily be automatically assumed following a breast cancer diagnosis. In order to appropriately determine a patient’s ovarian cancer risk (and thus their eligibility or indication for oophorectomy), a full evaluation should be performed including genetic testing, gathering of family history, pelvic imaging if the patient is symptomatic, and hormone receptor status of breast cancer. Common clinical scenarios in which oophorectomy may be indicated are as follows:

- Germline pathogenic variant that carries an increased risk of ovarian cancer (such as BRCA1/2 but also including PALB2, BRIP1, RAD51C/D, and Lynch syndrome genes)
- Strong family history of ovarian cancer (particularly a first-degree relative with ovarian cancer)
- Premenopausal patient who wishes or needs to undergo oophorectomy for the purposes of ovarian ablation in order to suppress ovarian function and to take endocrine therapy
- Presence of pelvic mass is concerning for malignancy, for which practitioners should consult a gynecologic oncologist

Unfortunately, randomized controlled trials evaluating utility of pelvic ultrasound and CA125 do not demonstrate decreased mortality utilizing these screening modalities in the general population [23]. Although still utilized among those who are considered high-risk, they should be interpreted with some caution given the possibility of false positives.

### Bone Health

Bone health is also a component of routine gynecologic and primary care. As per USPSTF and ACOG, recommendations for the general population include screening for osteoporosis in postmenopausal patients at age 65 and older using bone mineral density (BMD) testing with DEXA (dual x-ray absorptiometry) scans [24, 25]. For postmenopausal patients younger than 65 years old or who have other risk factors identified after risk assessment, screening with BMD testing is also recommended. Although there is no clear consensus on screening schedule in postmenopausal patients under 65, recommendations include repeating BMD testing no sooner than every 2 years [24]. Vitamin D, calcium, and weight-bearing exercise become extremely important [26]. Bisphosphonates should be considered in cases of osteoporosis.

In the setting of breast cancer, patients may be at increased risk of osteoporosis and osteopenia due to treatment-related factors that decrease circulating estrogen such as premenopausal ovarian suppression, premature ovarian failure due to chemotherapy, or use of aromatase inhibitors (AIs) [27]. Recommendations from the American Society of Clinical Oncology include periodic BMD testing while on AIs [28]. Additionally, NCCN recommendations include baseline evaluation of BMD for patients starting AIs who may have additional risk factors for osteoporosis [10].

There are some considerations regarding bone health among Black breast cancer survivors. Black people have generally lower baseline risk of osteoporosis compared to their White counter-parts, but similar fracture risks after an osteoporosis diagnosis [29]. However, given that Black patients are more likely to be diagnosed with breast cancer before 40 years old compared to other races [30], these patients may require suppression of ovarian function for endocrine therapy and/or may experience loss of ovarian function due to chemotherapy [31–35]. All of these factors could lead to loss of bone mineral density.

Regarding endocrine therapy, tamoxifen and AIs have differing effects on bone health. While tamoxifen has been associated with decreased bone density in premenopausal patients, other studies suggest no changes in BMD in postmenopausal patients on tamoxifen or even some bone protection [36–38]. Conversely, AIs have been shown to cause increased bone mineral loss and greater risk of fracture [39]. One Swedish study showed an increased risk of fracture among AI users compared to patients on tamoxifen (HR 1.48; 95% CI 0.98–2.22) and a higher risk of death for those hospitalized for fracture compared to those without fracture (HR 1.83; 95% CI 1.50–2.22) [40]. Another study showed that osteoporosis and fractures represented a common comorbidity for breast cancer survivors on endocrine therapy. This risk was higher with AI use compared to tamoxifen. Of note, only 1.7% of this particular total study population was Black and the majority of AI users were White [41]. Further research in this area among Black populations is warranted.

### Other Considerations for Primary Care

While the routine gynecologic visits for breast cancer survivors may not necessarily differ for those of the Black community, there are certain considerations that practitioners should appreciate (particularly for those gynecologists who may also serve as the patient’s sole primary care provider). For instance, Black people are more likely to experience cardiovascular toxicity from breast cancer treatments such as left-sided radiation therapy, chemotherapeutic agents such as anthracyclines, and targeted drug therapies such as trastuzumab [42–44]. Moreover, early-onset breast cancer diagnoses (for which Black people are at increased risk) may require oophorectomy for ovarian ablation in certain instances or may undergo chemotherapy which could lead to premature ovarian failure. Both of these outcomes increase the risk of cardiovascular disease [45]. This fact, along with data demonstrating that Black communities carry a higher baseline risk of cardiovascular disease [46], should prompt providers to thoroughly adhere to screening for cardiovascular disease risk and evaluate further as needed.

## Tamoxifen Use

Tamoxifen is a selective estrogen receptor modulator (SERM) that can be used as adjuvant endocrine therapy for breast cancer patients. While tamoxifen can decrease breast cancer recurrence and mortality [47], medication side effects are common and can impact tolerability and adherence. These include hot flashes, night sweats, venous thrombo-embolism (VTE), stroke, vaginal discharge, ovarian cysts, and cataracts. Limited data suggest that pregnancy should be avoided while on tamoxifen due to possible teratogenic effects, so reliable contraception is recommended. While there is no clearly established data, a “washout” period is usually recommended following cessation of tamoxifen and conception [48].

A major side effect of tamoxifen includes abnormal uterine bleeding (AUB). Due to tamoxifen’s partial agonist/antagonist effect on the uterus, it can produce variable results including no changes in regular menses while inducing amenorrhea in others. However, a common side effect of tamoxifen is the formation of uterine polyps which can cause heavy bleeding or intermenstrual bleeding. Additionally, data suggests that tamoxifen can increase the risk of endometrial hyperplasia, endometrial cancer, and uterine sarcomas in postmenopausal patients [49•]. Of note, while some research suggests that this risk could extend to premenopausal patients [50], this data may be affected by categorization bias and more research in this area is required [51].

There is no routine screening recommended for patients on tamoxifen. Symptom monitoring should be encouraged. Evaluation is indicated for premenopausal patients who present with AUB (such as heavy bleeding or intermenstrual bleeding) or any postmenopausal patients with bleeding [49•]. Office hysteroscopy coupled with endometrial biopsy is often a preferred initial approach, as transvaginal ultrasound does not reliably reflect the endometrial lining due to tamoxifen-induced subepithelial stromal hypertrophy [52]. These changes cause the lining to appear abnormal, making it challenging to interpret even in the absence of pathology.

Estimated risk of endometrial cancer with tamoxifen use is two- to three-fold higher than the average risk population [53–55] and research suggests that the risk is dose-dependent [56]. Based on data from the National Surgical Adjuvant Breast and Bowel Project, the risk of endometrial cancer for postmenopausal patients on tamoxifen is estimated to be 1.6 per 1000 patient years versus 0.2 per 1000 patient years in the control group [55]. One interesting retrospective study examined endometrial biopsies on 361 breast cancer survivors on tamoxifen. Of the total participants, 65% had normal endometrium, while 31.9% had endometrial polyps, 1.7% demonstrated endometrial hyperplasia, and 0.8% were diagnosed with endometrial cancer. Furthermore, endometrial thickness of ≥ 10 mm or greater had high diagnostic potential for postmenopausal (but not premenopausal) patients [57]. Another retrospective study demonstrated that tamoxifen use was associated with higher risk endometrial cancer subtypes (such as grade 3 endometrioid, papillary serous, or clear cell), with this risk being associated with longer duration of medication use [58].

There is limited data regarding the gynecologic outcomes of Black breast cancer survivors on tamoxifen. One particular retrospective cohort study investigated symptom burden by race and its relationship to endocrine therapy adherence [59]. Using data from a large multidisciplinary cancer center, researchers used 9 major symptom clusters, one of which was gynecologic symptoms. These included vaginal itching, vaginal bleeding/discharge, vaginal dryness, reduced sexual enjoyment, interest, or performance, breast tenderness/discharge, new lump/mass, and menstrual pain/cramping. Black patients reported more moderate severity symptoms such as hot flashes, higher baseline gynecologic symptoms, and more GYN symptoms at 1-year follow-up compared to White patients. These symptoms also some-times resulted in medication nonadherence. Although Black patients had lower adherence as demonstrated in other studies [60, 61], Hu et al. suggest that they may have had higher adherence than White people if symptom burden as well as socio-demographics were similar [59].

Another study evaluated adherence to endocrine therapy stratified by race [62]. Black people more frequently reported nonadherence compared to White people (13.7% versus 5.2%, respectively; *P* < 0.001), although Black race was not significantly associated with discontinuation. Nonadherence was more likely to be related to tamoxifen use and was more common in people who did not report engaging in shared decision-making with their providers (*P* < 0.001 for both). Regarding symptoms, Black people were more likely than White people to report hot flashes and night sweats but were less likely to report sexual side effects such as vaginal dryness and dyspareunia.

While data point to forgetting to take pills, cost, and low perceived risk of cancer recurrence as contributors to medication nonadherence [62], the possible contribution to tamoxifen side effects should not be discounted. Although data is limited, it suggests that Black patients may experience more tamoxifen-related symptomatology compared to White patients. While data does suggest that lack of adherence to endocrine therapy could be a possible contributor of Black breast cancer-related mortality, it is still unclear if gynecologic symptoms specifically are the driver. Importantly, much of the existing data focuses primarily on hot flashes and night sweats as predominant tamoxifen-related symptoms impacting adherence, with little data on the impact and prevalence of AUB.

Unfortunately, there is no clear solution to prevent gynecologic symptoms such as AUB or predict the nature of bleeding which the patient may experience. Hysterectomy is definitive treatment for AUB. However, routine hysterectomy is not indicated for patients taking tamoxifen unless in situations of atypical endometrial hyperplasia or endometrial cancer [63]. Black people may have other risks for AUB, for which the compounded use of tamoxifen remains unclear. Other pathologies that may cause AUB are discussed in the next section. Oncologists should consider these factors in decision-making regarding breast cancer treatment options.

## Abnormal Uterine Bleeding

AUB is described as any bleeding from the uterus in a non-pregnant person that is abnormal in regard to frequency, volume, or duration [64, 65]. Etiology can be structural (such as polyps or leiomyomas) or non-structural causes (such as coagulopathy or ovulatory dysfunction). Leiomyomas (also known as fibroids) are benign smooth muscle tumors that can cause heavy persistent bleeding, abdominal pain, or bulk symptoms which could interfere with micturition, defecation, and fertility.

Data suggest that Black patients are two- to threefold more likely to be diagnosed with fibroids, have fibroids at earlier ages, have a larger quantity of fibroids, and have larger volume of fibroids compared to White patients [66–70]. Treatment can vary based on the size and interval growth, as well as the severity of patient symptoms and fertility plans. The large majority of pharmacologic therapy for fibroids (and AUB in general) is hormonal. Oral contraceptives (with estrogen and progesterone or with progesterone alone), along with progesterone-containing intrauterine devices, are often first-line options for patients with AUB and fibroids [71]. However, these are generally contraindicated in patients with a history of breast cancer which could make management of AUB challenging for this population.

Non-hormonal options for treating AUB in breast cancer survivors are limited, but may include non-steroidal anti-inflammatory drugs (NSAIDS) and tranexamic acid (TXA). GnRH agonists have demonstrated utility in shrinking the size of large fibroids, but this is only temporary and usually performed in preparation for myomectomy [71]. Surgical interventions could include endometrial ablation, uterine artery embolization, and myomectomy (which can be hysteroscopic, laparoscopic, or abdominal depending on the fibroid location and size). While endometrial ablation can be a less invasive surgical option for patients with AUB who are ineligible for hormonal therapy, this should be approached with caution in breast cancer survivors on tamoxifen, as subsequent endometrial sampling after endometrial ablation becomes challenging and less reliable [72].

Hysterectomy provides definitive management but also entails major surgery with its possible increased risks of bleeding, need for transfusion, infection, and damage to adjacent organs. It also results in inability to serve as a gestational carrier and potential long-term effects on pelvic floor support. Black people are 2.4 times more likely to undergo hysterectomy and 6.8 times more likely to undergo myomectomy for fibroids, along with having higher likelihood of preoperative anemia and need for blood transfusion [69].

While most AUB is benign, further evaluation may be indicated based on presentation, age, and other factors. All postmenopausal bleeding requires further evaluation to rule out malignancy. The first initial step includes pelvic ultrasound which carries a > 99% negative predictive value for an endometrial thickness ≤ 4 mm in the setting of postmenopausal bleeding [73]. However, Black communities demonstrate a higher prevalence of fibroids [74] as well as an increased prevalence of nonendometrioid cancer (which may be less likely to present with diffusely thickened endometrium but rather with focal lesions) [75, 76]. This could alter interpretation of the endometrial lining. A simulated retrospective cohort utilizing data from the Surveillance, Epidemiology, and End Results (SEER) national cancer registry demonstrated a fivefold underdiagnosis of endometrial cancer in Black people when utilizing standard cutoffs for endometrial thickness. Authors suggest that endometrial cancer may be undersampled and missed due to increased prevalence of fibroids and nonendometrioid cancer subtype, which may contribute to racial health disparities [77••]. These findings should be considered by gynecologic providers in their assessment of Black patients with AUB.

While endometrial cancer affects Black and White people similarly (28.1 versus 27.8 per 100,000, respectively, which is not corrected for hysterectomy rates), the mortality rate for Black people is nearly double that of White people (9.0 versus 4.6 deaths per 100,000, respectively) [21]. Among the largest disparity gap of any cancer, the 5-year relative survival is 63% in Black people compared to 84% in White people. While data suggest that a predominant driving factor in the Black community is obesity, other factors such as tamoxifen use and pathogenic germline mutations could also pose risk [1, 21]. Furthermore, some recent data suggests links between endometrial cancer and Black hair products [78].

More research in this area is warranted to determine the independent effects of these factors as well as the compounded risk. Given the overlap between Black people’s increasing population endometrial cancer risk and possible tamoxifen use in the setting of breast cancer, providers should maintain a low threshold to evaluate patients with AUB to ensure benign pathology in the setting of multiple risk factors.

## Treatment of Menopausal Symptoms

### Vasomotor Symptoms

Hot flashes and night sweats (generally referred to as vasomotor symptoms or VMS) are commonly experienced in menopause due to loss of ovarian function, typically occurring between the ages of 51–52 in the U.S. Studies suggest that Black communities at baseline experience greater VMS at the time of menopause compared to other races [79, 80]. The Study of Women’s Health Across the Nation (SWAN)—a multisite, multiracial longitudinal study on the menopausal transition—represents one of the largest and most inclusive of Black participants (*n* = 935 of 3302 total). Black people had the highest prevalence and longest duration of VMS, as well as shorter sleep duration and less efficient sleep in menopause compared to White people [81]. Black people also had a statistically significant earlier median age at menopause compared to White people [82].

While VMS may result from natural menopause that is time concurrent with a breast cancer diagnosis, it can also result from the following:

- Chemotherapy (chemotherapy-induced menopause) due to gonadotoxic agents such as cyclophosphamide which could induce a temporary or permanent menopause
- Gonadotropin-releasing hormone (GnRH) agonist use in premenopausal patients to suppress ovarian function in preparation for or during chemotherapy to provide some level of ovarian protection
- GnRH use in premenopausal patients for ovarian suppression in the setting of endocrine therapy
- Surgical ovarian ablation (or surgically-induced menopause) for which the patient has undergone oophorectomy for ovarian ablation in order to take aromatase inhibitors (due to factors such as patient preference, inadequate ovarian suppression with GnRH agonists, or medication allergy)
- Side effect of tamoxifen or aromatase inhibitor use

In the setting of breast cancer, Black populations may have certain unique risk factors that increase their likelihood of experiencing vasomotor symptoms. For instance, Black breast cancer patients are more likely to undergo neoadjuvant chemotherapy (NACT) due to higher likelihood of aggressive disease. Cyclophosphamide, a known gonadotoxic agent, is frequently associated with temporary or permanent ovarian failure, which may subsequently increase vasomotor symptoms. While Black people demonstrate higher rates of TNBC (19% of total cases of Black people) which may require chemotherapy, there are still a large portion of breast cancer survivors who have luminal A types (57% of all Black people) [3], for which endocrine therapy could be indicated and for which VMS can be a side effect [59, 62].

Unfortunately, the mainstay of treatment for VMS includes systemic estrogen-based therapies. As this is contraindicated in breast cancer, other therapies should be explored. Selective serotonin reuptake inhibitors (SSRI) and serotonin norepinephrine reuptake inhibitors (SNRI) (such as venlafaxine), gabapentin, clonidine, and oxybutynin are commonly used non-hormonal options [83] for breast cancer survivors and can be trialed through shared-decision making and discussion of side effects. As of 2023, fezolinetant (Veozah) is a neurokinin 3 receptor antagonist approved by the U.S. Food and Drug Administration for the treatment of moderate to severe hot flashes [84]. However, side effects include elevations in liver transaminases, for which it is contraindicated in patients with cirrhosis, severe renal impairment, and concurrent use of CYP1A2 inhibitors [85]. Acupuncture and black cohosh represent alternative therapies with little data showing efficacy and with some data demonstrating interactions between black cohosh and tamoxifen that may decrease medication efficacy [86]. Globally, there is a dearth of data regarding racial differences in the treatment of vasomotor symptoms among breast cancer survivors. This is an area in which more research is required to better determine efficacy.

### Genitourinary Syndrome of Menopause (GSM)

GSM can encompass a variety of symptoms related to decreased estrogen levels, including vaginal dryness, burning, itching, dysuria, frequent urinary tract infections, and dyspareunia. This is thought to be due to vaginal atrophy, which causes decreased elasticity and lubrication of vaginal mucosa leading to painful or bothersome genitourinary symptoms [87].

Over-the-counter vaginal lubricants and moisturizers can help with vaginal dryness (such as coconut oil, vitamin E, water-, silicone-, or polycarbophil-based products, and hyaluronic acid). Topical lidocaine can also address dyspareunia specific to penetration [88]. However, vaginal atrophy is best treated with vaginal estrogen. Based on ACOG Clinical Consensus, if patients have failed non-hormonal therapy, low-dose vaginal estrogen can be used in breast cancer survivors [89]. While data do not suggest a significantly increased risk of breast cancer with vaginal estrogen due to smaller theoretical systemic absorption than oral estrogen, patients should still be made aware of theoretical breast cancer risks with any hormonal therapy and shared decision-making should be employed [90, 91]. Furthermore, concurrent use of vaginal estrogen in the setting of aromatase inhibitors is generally avoided due to data suggesting potential increased recurrence risk [92]. In all cases, gynecologic providers should communicate with the patient’s oncology team if conservative measures have failed and if vaginal estrogen is indicated.

Other treatments could include vaginal dehydroepian-drosterone (DHEA) and testosterone, which may improve dyspareunia and improve vaginal epithelium. However, the long-term impact of these therapies is still being studied. Ospemifene is a selective estrogen receptor modulator that is FDA approved for vaginal atrophy [87, 89]. Data in the general population are promising [93, 94], along with data that do not demonstrate increased recurrence rates in breast cancer survivors (although not FDA recommended in breast cancer survivors due to lack of data) [95]. Careful consideration must be given due to ospemifene’s agonist effects on endometrial tissue and increased risk of VTE [96]. Vaginal laser therapy is also being explored as a technique to stimulate remodeling and regeneration of vaginal tissue [89]. All of these therapies require additional research in breast cancer populations. None of them have been specifically studied in Black populations to date so their particular effects (if any) in this population remain unclear.

## Sexual Health and Libido

Breast cancer can have profound effects on sexual health and libido. Some of this may be related to physiologic changes such as vaginal atrophy-related dyspareunia from endocrine therapy or ovarian suppression. Medication side effects could also play a role; for instance, decreased libido could occur as a result of venlafaxine use (which could be indicated for hot flashes or depression, both of which may be relevant in the setting of breast cancer). Studies on testosterone have not demonstrated an improvement in libido for breast cancer survivors to date [97]; furthermore, the long-term impact of systemic testosterone in this community is unclear.

However, some of these symptoms could also be related to changes in identity and self-perceived femininity. Side effects of breast cancer treatment such as chemotherapy-induced hair loss (CIA) or mastectomy can be disfiguring and may impact feelings of self-worth. These factors could impact Black people disproportionately—as they may be more likely to require NACT and subsequently undergo CIA [98], and may be less likely to undergo breast reconstruction following mastectomy [99–102]. The long-term psychological impact of these outcomes has not been well-studied in this group. Gynecologists should work with patients and their oncologists to better identify changes in sexual health. While some issues can be addressed pharmacologically with vaginal lubricants and/or vaginal estrogen, the role of therapists, psychiatrists, and sexual counselors can and should be explored as well.

## Reproductive Considerations for Early-Onset Breast Cancer Survivors

### Contraception

The most commonly used contraception for AFAB people in the U.S. is hormonally based. However, data suggests that Black people in the general population have lower utilization of the hormonal contraceptive pill compared to White people, but similar use of condoms and long-acting reversible contraceptives [103].

As hormonal therapy is contraindicated in breast cancer, options may be greatly limited. This has implications for Black breast cancer survivors who are more likely than those of other races to have early-onset breast cancer, for which contraception may still be indicated to prevent pregnancy. The copper intrauterine device (IUD) represents a reliable, long-acting, and reversible form of contraception, with a failure rate of 0.8% [104]. However, common side effects include dysmenorrhea and menorrhagia. This should be taken into consideration for some Black patients who may already experience AUB due to structural factors such as fibroids. Condoms, cervical caps, and diaphragms with spermicide are other options although they are all associated with higher failure rates [105•]. Permanent contraception includes bilateral tubal ligation, bilateral salpingectomy, and vasectomy. Some breast cancer patients may be planning to undergo oophorectomy for ovarian ablation or risk-reduction due to a pathogenic germline mutation. While these patients will be inadvertently sterilized, oophorectomy is not recommended for sterilization alone in this population due to other issues such as increased cardiovascular mortality and bone thinning that cannot be mitigated with hormone therapy.

There are few studies examining contraception choices among Black breast cancer survivors in the United States. One study showed that breast cancer survivors remained sexually active during their cancer care and that condoms were the most frequently utilized contraceptive method [106]. However, of 150 total participants, only 4 were Black. More data are needed in this area to determine the contraceptive needs and possible barriers of Black early-onset breast cancer survivors.

### Assisted Reproductive Technology

The option of assisted reproductive technology (ART) should be discussed with all premenopausal breast cancer patients. These interventions could include oocyte cryopreservation prior to chemotherapy, ART following breast cancer treatments, or preimplantation genetic testing (PGT) for individuals with known pathogenic germline mutations. Notably, data suggest that Black patients are less likely to utilize ART overall [107]. These findings are concerning among a population already experiencing higher rates of early-onset breast cancer as well as higher likelihood of needing NACT which can be gonadotoxic [105•]. One population-based cohort study demonstrated that of 36,468 people with invasive breast cancer between the ages of 18–45, only 206 (0.56%) utilized ART. Of note, non-Hispanic Black and Hispanic patients were less likely than White patients to utilize the service (PR = 0.31; 95% CI 0.21–0.46) [108]. This represents an alarming and unacceptable racial disparity. It is imperative that practitioners offer this service to all premenopausal breast cancer survivors. As cost may also pose a barrier, more advocacy is needed for financial subsidies for fertility preservation as a component of comprehensive breast cancer care [109].

## Gender Diverse Populations

Most initiatives regarding breast cancer awareness, screening, and treatment primarily focus on AFAB people. However, gynecologic care may include transgender patients. Although breast cancer among people assigned male at birth (AMAB) is rare (< 1% of the general population), Black AMAB people have a higher incidence of “male” breast cancer compared to other racial groups [110–112], along with a higher incidence of TNBC [112].

Breast cancer prevalence and outcomes among transgender individuals are unclear. One study of transgender veterans in the U.S. showed an incidence rate of 20.0/100,000 person-years (regardless of hormonal therapy use) [113]. To date, the impact of exogenous estrogen taken for gender affirmation on breast cancer risk for transgender women is unclear [114]. However, increased concentrations of prediagnostic endogenous estradiol have been found to be associated with breast cancer in AMAB people [115]; furthermore, a retrospective study from the Netherlands demonstrated a 46-fold increased breast cancer risk among transgender women (95% CI 27.2 to 75.4) compared to cisgender men [116]. While screening recommendations for those with enhanced breast tissue following exogenous hormone administration have not been established, some organizations have proposed biennial screening mammography starting at age 50 for transgender women who have had at least 5–10 years of feminizing hormone therapy [114, 117]. There are no established guidelines for transgender females taking estrogen for gender affirmation with concurrent breast cancer diagnosis.

There is a dearth of data on the long-term effects of testosterone on breast cancer risk for transgender men. Additionally, there is the possibility of discrepancies between gender-affirming chest surgery and risk-reducing mastectomy that should be considered [118, 119]. The amount of remaining breast tissue could impact screening and inform subsequent cancer risk. There is little data on breast cancer outcomes in Black transgender individuals, as well as gender-diverse individuals as a whole. This area warrants further research.

## Genetic Testing

Genetic counseling and testing (GCT) represents the cutting edge of precision medicine. GCT can identify those at increased baseline risk for breast cancer who may qualify for intensive breast surveillance and whose risk for other cancers may be increased. Most breast cancer survivors have already broached the subject of genetic testing (or at least their eligibility for genetic testing) with an oncology provider somewhere along the cancer care pipeline. However, a prior review of one’s family history by a medical practitioner should not always be assumed. Primary care providers are considered “frontline” in identifying those for whom genetic testing may be indicated [120]. Risk assessment should also ideally occur prior to a cancer diagnosis, with ACR suggesting that all Black patients under risk assessment for breast cancer by age 25 [11••].

While there is still ongoing debate regarding universal testing for all patients following a breast cancer diagnosis [121–123], the NCCN genetic testing guidelines present specific criteria based on personal and family history [18]. It is critical for gynecologists to address family cancer history and confirm prior evaluation for genetic testing eligibility. If this has not already been performed, it should be reviewed as a component of general gynecologic care for all patients. A thorough family cancer history includes all family members in both maternal and paternal lineages with cancer, type of cancer, age at diagnosis, and age at death or noting if the person is still living. Red flags include (but are not limited to) family members with TNBC, ovarian cancer, pancreatic cancer, male breast cancer, multiple family members with breast cancer, breast cancers ≤ 50 years old, or patients of Ashkenazi Jewish ancestry. Identifying a person with a pathogenic germline mutation could have significant medical implications for subsequent breast cancer screening, breast surgical decision-making, and other possible pharmacologic or surgical risk-reducing options for both breast cancer and other cancers. It could also have implications for cascade testing among other family members [124].

Data suggest that GCT is globally underutilized among Black populations [125–127]. There are several possible reasons for this, including medical mistrust, fear of carrying a harmful gene mutation, concerns about genetic discrimination, costs, and lack of information [128–131]. However, some data also suggest that provider-centered barriers may play a role. One study noted that a testing barrier observed among a cohort of African American early-onset breast cancer survivors included lack of GCT referral by healthcare providers [132]. Another study showed disparate referral rates between White and Black breast cancer survivors (92.7% versus 75.7%, respectively), even though there was no significant difference in uptake of GCT by race once referred [133].

Testing results among Black communities may reflect similar prevalence of germline mutations (such as BRCA1/2) as compared to White communities [134–136], with the exception of the BRCA1 c.815_824dup founder mutation noted particularly among Black populations in the Southern U.S. and Caribbean [137, 138]. However, due to lack of inclusion in genome-wide association studies (GWAS) [139] and genomic research as a whole, Black people are more likely to have variants of unknown or uncertain significance (VUS) when undergoing genetic testing [140–146]. These represent equivocal, non-actionable results that are monitored over time for change.

There is a need for more culturally appropriate outreach to Black communities to better address concerns of medical mistrust and to increase uptake of GCT. Concurrently, providers should also be cognizant of self-bias that may impact the GCT referral pipeline. More robust genomic research among communities of the African diaspora is also needed to improve classification of VUS.

## Conclusion

Breast cancer is prevalent nationwide across all races and ethnicities. However, Black patients suffer from disproportionate mortality related to breast cancer, as well as other breast cancer-related health disparities. In order to provide quality care, a multidisciplinary approach must be employed. It is important for gynecologic providers to be well-versed on the intersections of breast cancer and gynecologic care. This is critical as gynecologic practitioners have an incredible presence on the long-term healthcare of this patient population, often providing care that precedes a patient’s breast cancer diagnosis and extends beyond when a patient has been discharged from oncologic care. Clinical care must also be coupled with ongoing research and advocacy for this patient population. Strategies to improve breast cancer-related racial health disparities must be intentional and ongoing.

## Figures and Tables

**Table 1:** Strategies for addressing racial disparities in gynecologic care for Black breast cancer survivors

Racial health disparity	Strategic approach
Increased likelihood of AUB due to leiomyoma	Ensure appropriate evaluation of AUB to rule out endometrial cancer Increase research on tamoxifen outcomes in Black communities
Greater likelihood of early onset breast cancer, leading to ovarian suppression or ovarian insufficiency from treatment	Assess heart and bone health at routine gynecology visits
Increased menopausal symptoms	Offer non-hormonal therapy for vasomotor symptoms Consider vaginal estrogen as needed Address sexual health concerns Increase research on efficacy of menopausal treatments among Black people
Decreased utilization of ART	Increase referrals to REI providers Public and private advocacy for more ART subsidies for cancer patients
Low uptake of GCT	Routine collection, updating, and evaluation of family cancer history Increase referrals to GCT as indicated Address medical mistrust through culturally appropriate messaging and community outreach Increase cancer genetics research in Black communities

Abbreviations: *AUB*, Abnormal uterine bleeding; *HR*, Hormone receptor; *ART*, Assisted reproductive technology; *REI*, Reproductive endocrinology and infertility; *GCT*, Genetic counseling and testing
